# Supplementary material for: Relationship between ideology and language in the Catalan independence context
Source: Sci Rep. 2019 Nov 20;9:17148. doi: 10.1038/s41598-019-53404-x (PMC6868228; doi:10.1038/s41598-019-53404-x)
Supplement: Supplementary file 1 — Supplementary Information [file 41598_2019_53404_MOESM1_ESM.pdf]

Supplementary Information

Relationship between ideology and language in the Catalan independence context

Julia Atienza-Barthelemy[1], Samuel Martin-Gutierrez[1], Juan C.Losada[1], and Rosa M. Benito[1,*]

[1]Grupo de Sistemas Complejos, Escuela Técnica Superior de Ingeniería Agronómica, Alimentaria y de Biosistemas, Universidad Politécnica de Madrid, Av. Puerta de Hierro, 2, 28040 Madrid, Spain.
[*]To whom correspondence should be addressed. E-mail: rosamaria.benito@upm.es

ABSTRACT

The supplementary Information of "Relationship between ideology and language in the Catalan independence context" is presented below

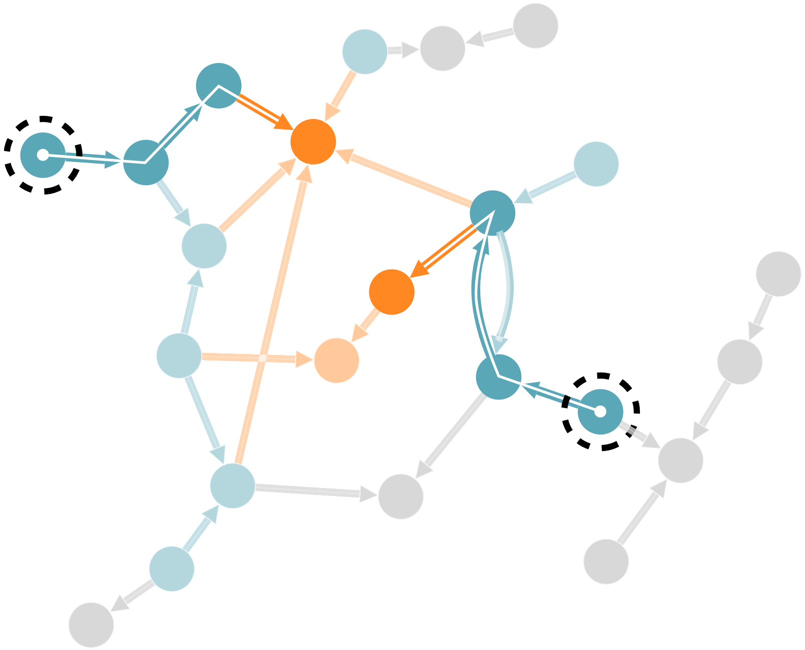

Figure S1. Scheme of listeners selection. Listeners users (blue) are those which have a directed path to an elite users (orange). The gray users are those for whom there is no directed path from the user to any elite user. There are two examples of paths from two circled listeners to two elite users highlighted in white.

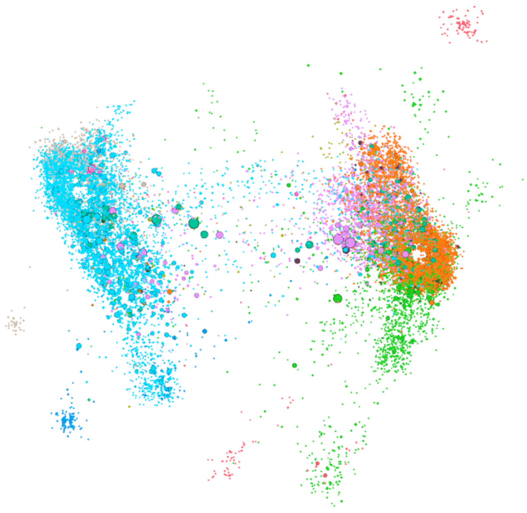

Figure S2. Retweet network of users connected with the elite users in a Catalan independence conversation, i.e., the listeners users. Each node is a user and the directed edges are the retweets between them.

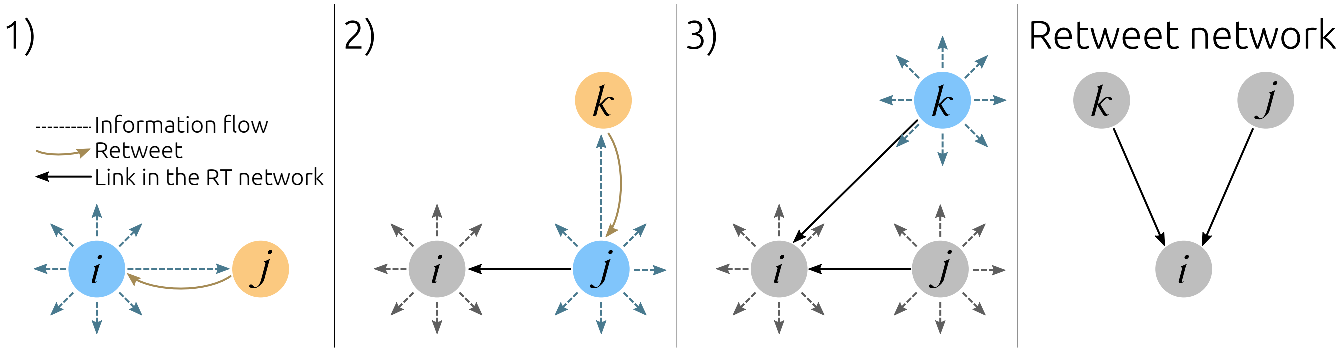

Figure S3. Schema that shows the method used to build the retweet networks. In 1), user j retweets a tweet originally posted by user i which means that j is being influenced by i's ideas. In 2), user k reads the tweet retweeted by j and retweet it again. In 3) the three users are information transmitters and in 4) the resulting retweet network is shown.

Numerical computation of the entropy of a continuous distribution

Since the opinion index is a continuous variable, its entropy should be computed using its probability density function (PDF). If $f(x)$ is the PDF of the continuous random variable x with support χ, the corresponding entropy would be:

$$h(f) = -\int_{\chi} f(x) \log f(x) dx \tag{1}$$

Here, a difficulty arises, because there is no analytical expression for the PDF of the opinion index. We have tackled this issue by computing the histogram of the opinion index, which yields a list of equally spaced intervals of the opinion index with their associated probability density. Then, we have performed a cubic interpolation taking the center of the intervals and the probability density values and we have used it to calculate (1) numerically. We have assessed the correctness of this methodology by verifying that the interpolated PDF is correctly normalized and that the resulting entropies are consistent when we change the number of bins employed to compute the histogram (we have tested values from 10 to 1000).
